# A Greater Flavonoid Intake Is Associated with Lower Total and Cause-Specific Mortality: A Meta-Analysis of Cohort Studies

**DOI:** 10.3390/nu12082350

**Published:** 2020-08-06

**Authors:** Mohsen Mazidi, Niki Katsiki, Maciej Banach

**Affiliations:** 1 Department of Twin Research and Genetic Epidemiology, Kings College London, London SE1 7EH, UK; 2 First Department of Internal Medicine, Division of Endocrinology and Metabolism, Diabetes Center, Medical School, AHEPA University Hospital, 546 21 Thessaloniki, Greece; nikikatsiki@hotmail.com; 3 Department of Hypertension, Chair of Nephrology and Hypertension, Medical University of Lodz, 93-338 Lodz, Poland; maciej.banach@icloud.com; 4 Polish Mother’s Memorial Hospital Research Institute (PMMHRI), 93-338 Lodz, Poland; 5 Cardiovascular Research Centre, University of Zielona Gora, 65-046 Zielona Gora, Poland

**Keywords:** flavonoid intake, total mortality, cancer mortality, cardiovascular disease mortality, meta-analysis

## Abstract

**Introduction:** The links between flavonoid intake and mortality were previously evaluated in epidemiological studies. The aim of the present study was to perform a systematic review and meta-analysis of cohort studies evaluating the link of flavonoid consumption with total and cause-specific mortality. **Methods:** Prospective cohort studies reporting flavonoid intake and mortality data published up to 30th April 2019 (without language restriction) were searched using PubMed, Scopus and EMBASE database. Generic inverse variance methods and random effects models were used to synthesize pooled and quantitative data. Sensitivity analysis was also performed by a leave-one-out method. **Results:** Overall, 16 articles met the inclusion criteria (nine studies were performed in Europe, five in the USA, one in Asia and one in Oceania); a total of 462,194 participants (all adults aged >19 years) with 23,473 mortality cases were included in the final analysis. The duration of follow-up ranged from 4.8 to 28 years. Most of the studies assessed flavonoid intake using food frequency questionnaires, whereas four studies used interviews and 1 study used 4-day food records. The meta-analysis showed that flavonoid consumption was inversely and significantly associated with total (relative risk (RR): 0.87, 95% confidence interval (CI) = 0.77–0.99) and cardiovascular disease mortality risk (RR: 0.85, 95%CI = 0.75–0.97), but not cancer (0.86, 95%CI = 0.65–1.14) mortality risk. These findings remained robust in sensitivity analyses. **Conclusions:** The present findings highlight the potential protective role of flavonoids against total and cause-specific mortality. These results support the recommendations for flavonoid-rich foods intake to prevent chronic diseases.

## 1. Introduction

Cardiovascular disease (CVD) remains the leading cause of death worldwide; however, in the high-income countries, cancer deaths have exceeded it [1]. The total number of deaths from non-communicable diseases increased from 2007 to 2017 by 22.7%; there were 7.61 million additional deaths estimated in 2017 (in comparison to 2007) [1]. The beneficial role of a healthy, balanced diet (i.e., rich in fruits and vegetables) on preventing CVD and cancer morbidity and mortality has been demonstrated in several large cohort studies [2,3].

Flavonoids present in foods may contribute to CVD protection [4]. Flavonoids are characterized by a shared skeleton of diphenylpropane, found in flavones, flavonols, flavanones, flavan-3-ols, etc., commonly present in vegetables, fruits, herbs and teas [4]. Flavonoids exert antioxidant properties and thus they may reduce plasma low-density lipoprotein (LDL) oxidation [5]. Furthermore, flavonoids may beneficially affect the vascular endothelium by inhibiting platelet aggregation and thus reducing the risk of clot formation [5]. However, available data on the association between flavonoid intake and all-cause, cancer and CVD mortality are limited and inconsistent [4,6,7,8,9,10,11,12,13,14,15,16,17,18,19,20,21]. In this context, with regard to the risk of total mortality, studies reported inverse [17], insignificant [4,9,11,16,18] or positive associations with flavonoid consumption [7]. For example, among 93,145 young and middle-aged women in the Nurses’ Health Study II (NHS-II), those in the highest fifth of flavonoid intake had an insignificantly lower total (risk ratio (RR): 0.92, 95% confidence interval (CI): 0.80-1.06), cancer (RR: 0.84, 95%CI: 0.67–1.04) and CVD (RR:0.83, 95%CI: 0.53–1.29) mortality risk compared with those in the lowest fifth [20].

A previous meta-analysis (*n* = 10 studies) showed a protective role of flavonoid intake in relation to total mortality (RR: 0.82, 95% 0.72–0.92), but no significant correlation with CVD mortality (RR: 0.85, 95%CI: 0.70–1.03) [21]. Regarding cancer mortality, data are also scarce with contradictory results: two studies reported a non-significant association [7,16], while another one supported a protective role for flavonoid intake [17]. Another meta-analysis (*n* = 15 cohort studies) found that flavonoid intake significantly decreased total and CVD mortality but did not investigate the association with cancer mortality [5].

In the present meta-analysis, we systematically reviewed current data from prospective studies on flavonoid consumption in relation to the risk of total, cancer and CVD mortality.

## 2. Methods

### 2.1. Literature Search and Study Selection

The Observational Studies in Epidemiology (MOOSE) guidelines were followed to design, conduct and report the present meta-analysis [22]. The primary exposure of interest was flavonoid intake, while the primary outcome of interest was alterations in cause-specific and total mortality subsequent to flavonoid consumption. Prospective cohort studies published up to 30 April 2019 (without language restriction) were searched in the EMBASE, PubMed and Scopus databases. The query syntax of the search is shown in Supplementary Table S1. We excluded duplicates, studies with participants aged ≤18 years at baseline and animal studies. Eligible studies were selected by using the predefined inclusion criteria of prospective cohort studies and original articles on the association of flavonoid intake and all-cause, cancer and CVD mortality. Furthermore, the reference list of eligible articles was searched and email correspondences with authors for additional data were performed, if needed.

### 2.2. Study Selection

**Figure 1:**
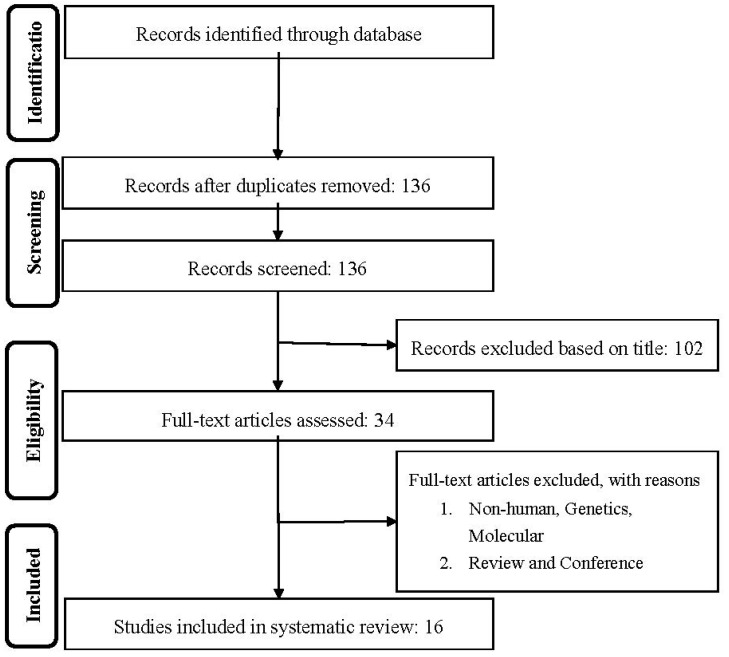
Flow chart of literature search for meta-analysis on flavonoid intake with total and cause specific mortality for the studies selection.

First, duplicates were removed, and then titles and abstracts were screened by two reviewers (MM and NK) who were blinded to the qualifications, names or the institutional affiliations of the study authors to avoid bias. Between these two reviewers, agreement was excellent (Kappa index: 0.91; *p* < 0.001), and any disagreements were resolved between the reviewers before the selected articles were retrieved (Figure 1 shows the flow chart of study selection).

Studies were included if they met all the following criteria: (1) the primary exposure of interest was flavonoid consumption; (2) the studies were population-based cohort studies, reporting total, CVD and cancer mortality; (3) hazard ratio (HR), RR or odds ratio (OR) estimates with 95%CI adjusted for multivariable parameters were provided or were able to be calculated; and (4) original articles with full texts in English.

We excluded: (1) letters, reviews, comments, editorials, expert opinion papers, methodological articles, unpublished data or any publication lacking primary data and/or explicit method descriptions; (2) non-English papers; (3) studies other than population-based cohorts; and (4) studies in which HR, RR or OR estimates with 95%CI were not available or not able to be calculated.

### 2.3. Data Extraction

The full texts of the included studies were retrieved and screened for eligibility by two reviewers (Mohsen Mazidi, Niki Katsiki). The Newcastle–Ottawa Scale (NOS, Supplementary Table S2) was used to assess the quality of the included studies [23], considering dietary survey methods of flavonoid intake; representativeness of the exposed cohort; assessment of outcome; comparability of cohorts (adjustment for important confounders); duration and adequacy of follow-up. By evaluating selection, comparability and outcome, the study score ranged from 0 (highest degree of bias) to 9 (lowest degree of bias). Following methodological quality assessment, the 2 reviewers extracted data by using a purpose-designed data extraction form. They also independently produced a summary of the most important outcomes from each study. Any differences of opinion in relation to these summaries were resolved by consultation with a third reviewer (Maciej Banach). The first reviewer (Mohsen Mazidi) conducted any further necessary calculations on data, checked by the second reviewer (Niki Katsiki). The data extracted from each eligible study included: author, study name, year, country, references, mean age, men (%), the number of cases and participants, follow-up time (years), main confounders and outcomes.

### 2.4. Data Synthesis and Statistical Analyses

When study results were from different multivariable-adjusted models, the model with the majority of the confounding parameters was used for the present meta-analysis. Pooled RR, 95%CI and *p* value for heterogeneity were calculated with the random-effect model. RRs comparing the highest with the lowest flavonoid intake category were combined across studies to generate the summary associations. The *I*^2^ test [24,25,26] and *I*^2^ > 50% together with *p* (two-sided) < 0.05 indicated significant heterogeneity across studies [24,25,26]. A sensitivity analysis, excluding one study at a time, was conducted to examine whether the present results were affected by a single study.

### 2.5. Publication Bias

The presence of publication bias was evaluated by the visual inspection of Begg’s funnel plot asymmetry and Egger’s weighted regression tests. Furthermore, the Duval and Tweedie trim method was applied to adjust the analysis for the effects of publication bias [27]. The present meta-analysis was conducted with the use of the Comprehensive Meta-Analysis (CMA) V3 software (Biostat, Englewood, NJ, USA) [28].

## 3. Results

Overall, of the 34 eligible full articles, 16 articles met the inclusion criteria (Figure 1). An overview of the key characteristics of these 16 prospective cohort studies is shown in Table 1. A total of 462,194 participants, with 23,473 mortality cases were included in the final analysis. The duration of follow-up ranged from 4.8 to 28 years. Quality assessment results are shown in the Supplementary Table S3. All participants were adults (aged >29 years) at baseline. With regard to geographical region, seven studies were carried out in Europe [4,7,8,10,14,18,19], five in the USA [6,11,12,15,20] one in Asia [13] and one in Oceania [17]. Six studies included both male and female individuals [9,10,15,16,18,19], and nine studies included only male [4,6,7,8,14] or female [11,12,17,20] participants. In the majority of the studies, flavonoid intake was assessed by food frequency questionnaires [6,8,11,12,13,14,15,17,18,19,20], whereas four studies used interviews [4,9,10,16] and one study used 4-day food records [7].

### 3.1. Associations of Flavonoid Intake with All Cause, CVD and Stroke Mortality

**Figure 2:**
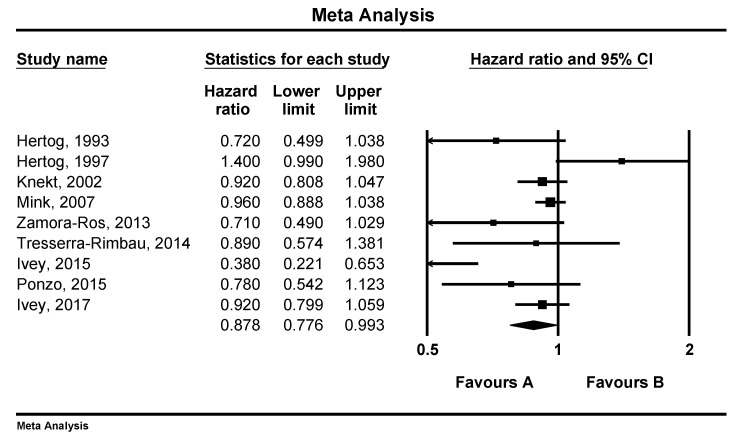
Forest plot of flavonoid intake and risk of total mortality.

**Figure 3:**
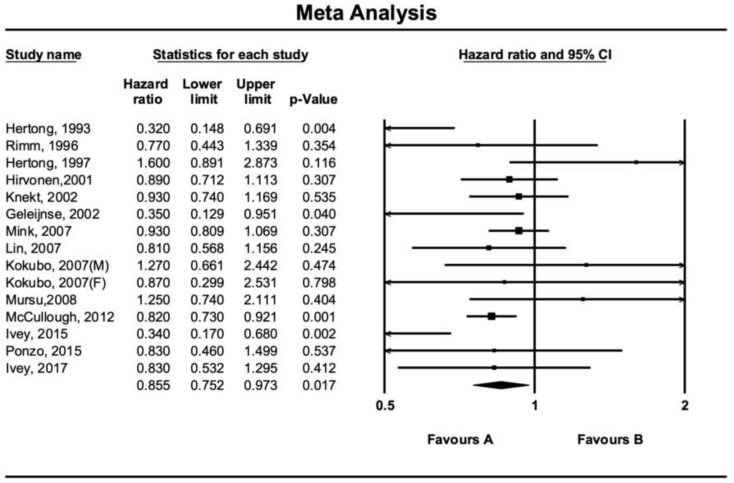
Forest plot of flavonoid intake and risk of cardiovascular disease mortality.

**Figure 4:**
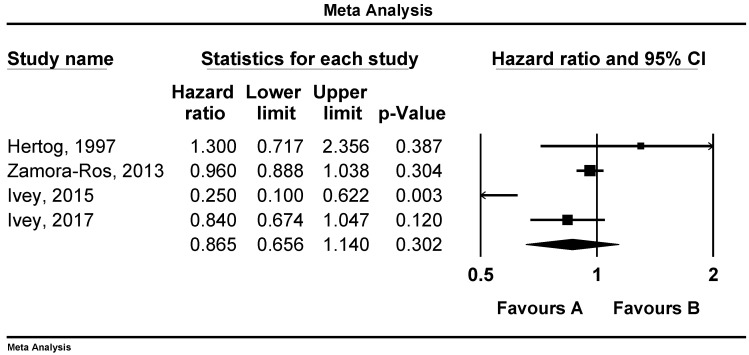
Forest plot of flavonoid intake and risk of cancer mortality.

There was a reverse and significant association between flavonoid consumption and all-cause mortality (RR: 0.87, 95%CI = 0.77–0.99, *p* = 0.039, *n* = 9 studies, Figure 2). Similar associations were observed between flavonoid intake and CVD mortality (RR: 0.85, 95%CI = 0.75–0.97, *p* = 0.017, *n* = 15 studies, Figure 3), but not with cancer mortality (RR: 0.86, 95%CI = 0.65–1.14, *p* = 0.302, *n* = 4 studies, Figure 4).

### 3.2. Sensitivity Analysis

In the leave-one-out sensitivity analyses, the pooled effect estimates remained similar for the effect of flavonoid intake on total (RR: 0.87, 95%CI = 0.77–0.99), CVD (RR: 0.85, 95%CI = 0.75–0.97) and cancer (RR: 0.86, 95%CI = 0.65–1.14) mortality.

### 3.3. Publication Bias

Visual examination of the funnel plot symmetry showed no publication bias for the association between flavonoid intake and total mortality. Similarly, the Egger’s linear regression (intercept = −1.30, two-tailed *p* = 0.170) indicated the absence of publication bias. After using the ‘trim and fill’ correction to adjust the effect size for potential publication bias, there were no potentially missing studies in the funnel plot. According to the ‘fail-safe N’ test, 20 studies would be needed for the RR to become non-significant (*p* > 0.05).

## 4. Discussion

In the present study, we evaluated the impact of flavonoid intake on total and cause-specific (CVD and cancer) mortality. Pooled data from observational prospective studies showed a significant and negative relationship between flavonoid intake and the risk of all-cause and CVD mortality.

Previous studies on flavonoid consumption and mortality have reported conflicting findings. Our results are in agreement with previous meta-analyses of randomized controlled trials (RCTs) and observational studies on flavonoids, suggesting that their intake might be protective against CVD, cancer and total mortality [5,29,30,31]. Furthermore, consuming fruits and vegetables (the main source of flavonoids) has been associated with a decreased risk of mortality in a meta-analysis [32]. Flavan-3-ols (from cocoa products and green tea), as well as anthocyanins (from berry fruits), were related to a reduced risk of CVD [33,34]. In addition, wine and beer (one of the main source of flavonoids) consumption have been reported to have a J-shaped relationship with CV-related outcomes; the degrees of potential protection by the phenolic or alcoholic content remain unclear [35]. Studies on soy products (rich in isoflavones) and mortality are scarce, but a few available investigations reported no significant association [36,37]. With regard to the impact of flavonoids on CV risk factors, meta-analyses of RCTs suggest that the benefits related to green tea and cocoa products (main sources of flavan-3-ols) was due to a reduction in LDL-C and improvements in endothelial function and insulin sensitivity [33,38]. Flavonoid intake has also been inversely associated with hypertension [39].

In contrast with our findings, another study (*n* = 93,145 young and middle-aged US women in the NHS-II) found an insignificant relationship between total-flavonoid intake and risk of all-cause mortality [20], a finding similar to the Iowa Women’s’ Health Study [11]. The authors of the NHS-II commented that the lack of any beneficial effect of flavonoids on CVD may be due to the cohort characteristics itself, namely the low CVD mortality rate in this middle-aged female population [20]. It should be noted that the complexity of the methods assessing flavonoid intake (total or specific), as well as regional differences in the food composition of dietary flavonoids [16], shaping the pattern of over 4000 different flavonoid compounds consumed on a daily basis [40], may have contributed to the inconsistent results. Furthermore, data on these differences in the food sources of flavonoids and the methods used to estimate total or specific flavonoid intake were not always available. A meta-analysis including 10 studies reported an inverse relationship between flavonoid intake with total mortality (RR: 0.82, 95% 95%CI: 0.72–0.92), with no significant association between flavonoid consumption and CVD mortality (RR: 0.85; 95%CI: 0.70–1.03) [21]. Of note, the number of studies evaluating CVD death in this meta-analysis was only five [22].

Several possible mechanisms through which flavonoids may benefit health and decrease mortality risk have been suggested [41,42,43]; the basic pathways involve their antioxidant and anti-inflammatory actions [44,45]. Flavonoids inhibit several processes implicated in disease progression, such as oxidative stress and inflammation, that are the main determinants of CVD [45]. Based on the in vitro data, flavonoids can decrease oxidative damage through free radical scavenging activity [46]. Furthermore, non-human studies reported that high flavonoid intake was related to low levels of established biomarkers of inflammation such as the nuclear factor kappa-B (NF-kB) and C-reactive protein [47]. We have previously reported a detrimental impact of inflammation and oxidative stress on cardiometabolic risk factors [48,49,50,51,52]. We suggested that flavonoids may also prevent CVD via (1) affecting endothelial function and vascular homeostasis with the production of factors that act locally in the vessel wall and lumen, e.g., prostacyclin, nitric oxide and endothelin; (2) exerting antifibrotic effects and regulating fibrinolytic factors, e.g., the tissue plasminogen activator and plasminogen activator inhibitor-1; and (3) affecting platelet aggregation and coagulation by influencing the production of adhesion molecules and inflammatory cytokines [44]. Nevertheless, theses mechanisms need to be further investigated in human studies.

Our non-significant results regarding the link between flavonoids intake and cancer mortality may be attributed, at least partly, to the small number of studies. However, flavonoids were reported to protect against cancer by a direct inhibition of oxidative stress and damage [53]. Moreover, flavonoids can exert anti-proliferative, anti-angiogenic and anti-metastatic properties by modulating several receptors and enzymes in several signal transduction pathways related to cellular proliferation, differentiation and apoptosis [53]. Overall, in vitro evidence supports the potential role of dietary flavonoids as protective compounds, but further observational studies and RCTs are needed to confirm the associations of flavonoid intake with all-cause, CVD and cancer mortality found in the present study, as well as to elucidate the underlying mechanisms of these links.

Consistent with our findings, previous meta-analyses highlighted possible negative associations between flavonoid intake and incidence of certain cancers, such as breast [29,54], prostate [55], lung [55], stomach and colorectal cancer [56], as well as smoking-related cancer [30]. In contrast, the link between total cancer mortality and flavonoid consumption has been rarely (four studies) investigated [7,16,17,20]. In one study, a strong inverse association between flavonoid intake and all-cancer mortality was observed (RR = 0.25, 95% CI: 0.10, 0.62) [17], but the other three studies showed non-significant correlations [7,16,20]. The protective effects of flavonoids on cancer risk may be explained by several mechanisms [57,58]. Although flavonoids, mainly isoflavones, are mostly characterized by their weak estrogenic activity, they also exert several other biologic actions that might influence cancer risk, such as antioxidant, anti-proliferative [59] and anti-angiogenic properties [60], together with the capacity to inhibit cytokines, growth factors, and several enzymes [61]. For example, flavonoids can effectively reduce various types of oxidants [62], subsequently reducing cancer risk. The direct antioxidant activities of dietary flavonoids may not be the only explanation for their protection against cancer mortality [63,64]. Another pathway involves the impact of flavonoids on the regulation of enzymes. Carcinogens are metabolized to more active forms by phase I enzymes such as the cytochrome P450 (CYP); the active forms are subsequently detoxified by phase II enzymes such as the UDP-glucuronyl transferase, glutathione S-transferase, and quinone reductase [65]. Flavonoids can inactivate phase I and phase II enzymes [65]. Therefore, flavonoids may exert anticancer effects, which could be influenced by established risk factors for cancer, such as alcohol consumption [66], energy intake, smoking, menopausal status and the use of hormonal replacement therapy [67].

Nevertheless, we cannot be certain that the inverse association between flavonoid consumption and CVD, cancer and all-cause mortality is casual, representing the effect of flavonoids only. In the present study, our model was adjusted for the main compounds of the diet, including fiber, protein, fat and carbohydrates. This adjustment attenuated our results but there was still an inverse link between flavonoid consumption, total, CVD and cancer mortality, thus highlighting the protective effect of flavonoids on the risk of death.

### Study Strengths and Limitations

The present study has strengths and limitations. A very low level of heterogeneity of the studies included to the meta-analysis was found, highlighting the validity of our results. Since the majority of the studies assessed flavonoid intake by food frequency questionnaires and flavonoids are present in several different foods, some misclassification of flavonoid consumption is inevitable. However, this misclassification could be non-differential and bias our results towards the null. Therefore, the pooled RR of the relationship between flavonoid intake and mortality could have been underestimated rather than overestimated. Secondly, we cannot rule out the possibility that unmeasured or residual confounding (characteristic of original studies) might have affected the observed associations, although we controlled for potential confounding variables with the use of RRs. Furthermore, the reliability of the available data may have been affected by (1) a small number of cases in some studies, possibly affecting the statistical power of the analyses; (2) a single baseline assessment of dietary intake, with a lack of specific information over time related to mortality; and (3) a lack of data on the associations between different flavonoid types and causes of mortality. Finally, some of the included studies involved specific groups of individuals, limiting the generalizability of these results to the general population [11].

## 5. Conclusions

Our findings highlighted the potential protective role of flavonoids against total, cancer and CVD mortality. These results further support the recommendations for flavonoid-rich foods intake to prevent cardiovascular diseases and cancers. Due to the potentially different effects of flavonoids, recommendations should also highlight the importance of dietary variety, including diverse flavonoid sources. Further research is required to establish the specific role of individual flavonoid classes as well as the quantities needed to be consumed to achieve health benefits.

## Figures and Tables

**Table 1:** Characteristics of the 16 prospective cohort studies included in the meta-analysis.

Author, Year and Reference	Country, Region/Cohort	Men (%)	Mean Age (Years)	Follow-Up Time (Years)	No. of Cases (Outcomes)	No. of Subjects	Outcomes (Mortality)	Main Confounders
Hertog (1993) [4]	Netherlands, Zutphen Elderly Study	100	65–84	5	43, 185	805	CHD, All-cause	Age, BMI, smoking, serum total and HDL-C, systolic blood pressure, intake of total energy, saturated fatty acids, cholesterol, alcohol, coffee, vitamin C, vitamin E, beta-carotene, dietary fiber, history of MI
Rimm (1996) [6]	USA, Health Professionals follow-up Study	100	40–75	6	140	34,789	CHD	Age, BMI, smoking, diabetes, profession, hypertension, high cholesterol levels, family history of CHD, intake of vitamin E, alcohol, dietary fiber, carotene and saturated fat
Hertog (1997) [7]	UK, Caerphilly study	100	45–59	14	131, 334	1900	IHD, All-cause	Age, BMI, smoking, systolic blood pressure, serum total cholesterol, history of IHD at baseline, social class, intakes of total energy, alcohol, fat, vitamin C, vitamin E, and beta-carotene
Hirvonen (2001) [8]	Finland, Alpha-Tocopherol, Beta-Carotene Cancer Prevention Study	100	50–69	6.1	815	25,372	CHD	Age, BMI, smoking, systolic and diastolic blood pressure, serum total cholesterol, HDL-C, diabetes, CHD history, marital status, educational level and physical activity
Knekt (2002) [9]	Finland, Finnish mobile clinic health examination survey	--	54.0 ± 10.6	28	681, 2085	9131	IHD, All-cause	Age, sex, geographic area, occupation, blood pressure, smoking, serum cholesterol, BMI, diabetes, intakes of energy, cholesterol, saturated fatty acids, fiber, vitamin E, vitamin C and beta-carotene
Geleijnse (2002) [10]	Netherlands, Rotterdam Study	38.3	>55	5.6	30	4807	MI	Age, sex, BMI, smoking, education level, daily intakes of alcohol, coffee, polyunsaturated fat, saturated fat, fiber, vitamin E, and total energy
Mink (2007) [11]	USA, Iowa Women’s Health Study	0	55–69	16	2316, 7091	34,489	CVD, All-cause	Age, BMI, waist-to-hip ratio, smoking, energy intake, marital status, education, blood pressure, diabetes, physical activity and estrogen use
Lin (2007) [12]	USA, Nurses’ Health Study	0	30–55	12	324	66,360	CHD	Age, BMI, current smoking, parental history of MI at an age <60 years, history of hypertension, hypercholesterolemia and diabetes, menopausal status, hormone replacement therapy, use of aspirin, multivitamin and vitamin E supplements, physical activity, alcohol consumption and total energy intake
Kokubo (2007) [13]	Japan, Japan Public Health Center-Based Study	25.8	40–59	12.5	1538	40,462	CVD	Age, sex, BMI, smoking, alcohol use, history of hypertension or diabetes, hypolipidemic drugs, education level, sports, dietary intake of fruits, vegetables, fish, salt, and energy
Mursu (2008) [14]	Finland, Kuopio Ischemic Heart Disease Risk Factor Study	100	42–60	15.2	153	1950	CVD	Age, examination years, BMI, systolic blood pressure, hypertension medication, serum HDL-C and LDL-C, serum TAG, maximal oxygen uptake, smoking, CVD in family, diabetes, alcohol intake, energy-adjusted intake of folate and vitamin E, total fat (percentage of energy) and saturated fat intake (percentage of energy)
McCullough (2012) [15]	USA, Cancer Prevention Study II Nutrition Cohort	38.8	69.5	7	2771	98,469	CVD	Age, sex, BMI, smoking, beer and liquor intake, history of hypertension and dyslipidemia, family history of MI, physical activity, energy intake, aspirin use, hormone replacement therapy (in women only)
Zamora-Ros (2013) [16]	Spain, EPIC-Spain cohort	38	29–70	13.6	1915	40,622	All-cause	Age, sex, BMI, education level, physical activity, smoking, lifetime alcohol consumption, total energy, vitamin C and fiber intake
Tresserra-Rimbau (2014) [18]	Spain, PREDIMED study	45.3	55–80	4.8	327	7172	All-cause	Age, smoking, BMI, diabetes, alcohol, total energy intake, physical activity, family history of CVD or cancer, aspirin use, antihypertensive drug use, use of oral hypoglycemic agents, insulin, other medication, intake of protein, saturated fatty acids, polyunsaturated fatty acids, monounsaturated fatty acids and cholesterol
Ivey (2015) [17]	Australia, Calcium Intake Fracture Outcome Age Related Extension Study	0	>75	5	78, 129	1063	CVD, All-cause	Age, prevalent CVD and cancer, overweight or obesity, low fruit and vegetable intake, physical inactivity, current cigarette smoking, alcohol consumption
Ponzo (2015) [19]	Italy, Local Health Units of the province of Asti	-	45–64	12	84, 220	1658	CVD, All-cause	Age, sex, BMI, education, living in a rural area, METs, fiber and saturated fatty acid intakes, alcohol intake, smoking, systolic and diastolic blood pressure, total and HDL-c, fasting glucose, CRP, statin and aspirin use
Ivey (2017) [20]	USA, Nurses’ Health Study II.	0	36.1	18	189, 1894	93,145	CVD, All-cause	Age, BMI, smoking, menopausal status, family history of diabetes, cancer and MI, multivitamin supplement use, aspirin use, race, diabetes, hypercholesterolemia, hypertension, physical activity, energy intake, alcohol consumption and the Alternative Health Eating Index (minus alcohol) score

CHD: coronary heart disease, CVD: cardiovascular disease, IHD: ischemic heart disease, BMI: body mass index, METs: metabolic equivalents, MI: myocardial infarction, HDL-C: high-density lipoprotein cholesterol, LDL-C: low-density lipoprotein cholesterol, TAG: triacylglycerol, CRP: C-reactive protein.

## Supplementary Materials

The following are available online at https://www.mdpi.com/2072-6643/12/8/2350/s1. Table S1: Full search terms and strategy for papers indexed in PUBMED, Table S2: Newcastle – Ottawa Quality Assessment Scale Cohort Studies, Table S3: Quality assessment of cohort studies on flavonoid intake, all-cause and cause-specific mortality.
