# Supplementary material for: A Greater Flavonoid Intake Is Associated with Lower Total and Cause-Specific Mortality: A Meta-Analysis of Cohort Studies
Source: Nutrients. 2020 Aug 6;12(8):2350. doi: 10.3390/nu12082350 (PMC7469069; doi:10.3390/nu12082350)
Supplement: Supplementary file 1 [file nutrients-12-02350-s001.pdf]

Supplementary Table 1. Full search terms and strategy for papers indexed in PUBMED

No	Concept	Search terms
**1**	**Flavonoids**	“flavonoids” [Mesh] OR “flavonols” [Mesh] OR “flavones” [Mesh] OR “anthocyanidins” [Mesh] OR “flavanones” [Mesh] OR “flavan-3-ols” [Mesh] OR “catechins” [Mesh] OR “proanthocyanidins” [Mesh] OR “quercetin” [Mesh] OR “myricetin” [Mesh] OR “kaempferol” [Mesh] OR “isorhamnetin” [Mesh] OR“apigenin” [Mesh] OR “luteolin” [Mesh]
**2**	**Mortality**	mortality[tiab] OR death*[tiab] OR dead[tiab] OR all-cause[tiab] OR all cause[tiab] OR fatal[tiab] OR event[tiab] OR nonfatal[tiab] OR non-fatal[tiab] OR Mortality[Mesh:NoExp] OR mortality[Mesh subheading]
**3**	**Cardiovascular**	cardiovascular[tiab] OR vascular[tiab] OR CVD[tiab] OR Cardiovascular Diseases[Mesh:NoExp]
**4**	**Combination**	#2 OR #3
**5**	**Combination Exposure And Outcome**	#1 AND #4
**6**	**Limit**	Rats[Mesh:NoExp]) OR Mice[Mesh:NoExp]) OR rat[Title/Abstract]) OR rats[Title/Abstract]) OR mouse[Title/Abstract]) OR mice[Title/Abstract]) OR vivo[Title/Abstract]) OR vitro[Title/Abstract])
**7**	**Limit**	#5 NOT #6

Supplementary Table 2. Newcastle – Ottawa Quality Assessment Scale Cohort Studies

Note: A study can be awarded a maximum of one star for each numbered item within the Selection and Outcome categories. A maximum of two stars can be given for Comparability.

Selection

1) <u>Representativeness of the exposed cohort</u>

a) truly representative of the average *healthy adults* in the community ★

b) somewhat representative of the average *healthy adults* in the community ★

c) selected group of users *e.g. nurses, volunteers, vegetarian*

d) no description of the derivation of the cohort

2) <u>Selection of the non-exposed cohort</u>

a) drawn from the same community as the exposed cohort ★

b) drawn from a different source

c) no description of the derivation of the non-exposed cohort

3) <u>Ascertainment of exposure</u>

a) secure record (*e.g. 7 day food diary)* ★

b) structured interview/≥ 2 *dietary recalls/diet history/ food frequency questionnaire validated for dairy components* ★

c) written self-report (*e.g. <2 dietary recalls/non-validated food frequency questionnaire or not reported whether food frequency questionnaire was validated*)

d) no description

4) <u>Demonstration that outcome of interest was not present at start of study</u>

a) yes ★

b) no

Comparability

1) <u>Comparability of cohorts on the basis of the design or analysis</u>

a) study controls for *age, sex, smoking, total energy intake, and body mass index* ★

b) study controls for any additional factor (*e.g. physical activity, alcohol intake, family history of diabetes, dietary factors*) ★

Outcome

1) <u>Assessment of outcome</u>

a) independent blind assessment (e.g. clinical diagnosis/complete medical information available). ★

b) record linkage/*medical record or validated self-report* ★

c) non-validated self-report

d) no description

2) <u>Was follow-up long enough for outcomes to occur</u>

a) yes/ *follow up period for outcome of interest is 10 years or over* ★

b) no

3) <u>Adequacy of follow-up of cohorts</u>

a) complete follow-up - all subjects accounted for ★

b) subjects lost to follow-up unlikely to introduce bias - small number lost *≤20%* follow-up, or description provided of those lost ★

c) follow-up rate <*80%* or no description of those lost

d) no statement

Supplementary Table 3. Quality assessment of cohort studies on flavonoid intake, all-cause and cause-specific mortality.

Studies	Selection				Comparability	Outcome			Total score
	Representativeness of the exposed cohort	Selection of the non-exposed cohort	Ascertainment of exposure	Outcome not present at start of study	Comparability of cohorts on the basis of the design or analysis	Assessment of outcome	Follow-up long enough for outcomes to occur	Adequacy of follow-up of cohorts	
Hertog (1993)(1)	C	A★	B★	A★	A★ B★	B★	A★	B★	8
Rimm (1996)(2)	C	A★	B★	A★	A★ B★	B★	A★	B★	8
Hertog (1997)(3)	C	A★	B★	A★	A★ B★	B★	A★	B★	8
Hirvonen (2001) (4)	C	A★	B★	A★	A★ B★	B★	A★	B★	8
Knekt (2002)(5)	C	A★	B★	A★	A★ B★	B★	B	B★	7
Geleijnse (2002) (6)	C	A★	B★	A★	A★ B★	B★	B	B★	7
Mink (2007) (7)	C	A★	B★	A★	A★ B★	B★	A★	B★	8
Lin (2007) (8)	C	A★	B★	A★	A★ B★	B★	A★	B★	8
Kokubo (2007) (9)	C	A★	B★	A★	A★ B★	B★	B	B★	7
Mursu (2008)(10)	C	A★	B★	A★	A★ B★	B★	A★	B★	8
McCullough (2012) (11)	C	A★	B★	A★	A★ B★	B★	A★	B★	8
Zamora-Ros (2013)(12)	C	A★	B★	A★	A★ B★	B★	B	B★	7
Tresserra-Rimbau (2014)(13)	C	A★	B★	A★	A★ B★	B★	B	B★	7
Ivey (2015)(14)	C	A★	B★	A★	A★ B★	B★	A★	B★	8
Ponzo (2015)(15)	C	A★	B★	A★	A★ B★	B★	B	B★	7
Ivey (2017)(16)	C	A★	B★	A★	A★ B★	B★	B	B★	7

References:

1. Hertog MG, Feskens EJ, Hollman PC, Katan MB, Kromhout D. Dietary antioxidant flavonoids and risk of coronary heart disease: the Zutphen Elderly Study. Lancet (London, England). 1993;342(8878):1007-11.
2. Rimm EB, Katan MB, Ascherio A, Stampfer MJ, Willett WC. Relation between intake of flavonoids and risk for coronary heart disease in male health professionals. Annals of internal medicine. 1996;125(5):384-9.
3. Hertog MG, Sweetnam PM, Fehily AM, Elwood PC, Kromhout D. Antioxidant flavonols and ischemic heart disease in a Welsh population of men: the Caerphilly Study. The American journal of clinical nutrition. 1997;65(5):1489-94.

4. Hirvonen T, Pietinen P, Virtanen M, Ovaskainen ML, Hakkinen S, Albanes D, et al. Intake of flavonols and flavones and risk of coronary heart disease in male smokers. Epidemiology (Cambridge, Mass). 2001;12(1):62-7.
5. Knekt P, Kumpulainen J, Jarvinen R, Rissanen H, Heliovaara M, Reunanen A, et al. Flavonoid intake and risk of chronic diseases. The American journal of clinical nutrition. 2002;76(3):560-8.
6. Geleijnse JM, Launer LJ, Van der Kuip DA, Hofman A, Witteman JC. Inverse association of tea and flavonoid intakes with incident myocardial infarction: the Rotterdam Study. The American journal of clinical nutrition. 2002;75(5):880-6.
7. Mink PJ, Scrafford CG, Barraj LM, Harnack L, Hong CP, Nettleton JA, et al. Flavonoid intake and cardiovascular disease mortality: a prospective study in postmenopausal women. The American journal of clinical nutrition. 2007;85(3):895-909.
8. Lin J, Rexrode KM, Hu F, Albert CM, Chae CU, Rimm EB, et al. Dietary intakes of flavonols and flavones and coronary heart disease in US women. American journal of epidemiology. 2007;165(11):1305-13.
9. Kokubo Y, Iso H, Ishihara J, Okada K, Inoue M, Tsugane S. Association of dietary intake of soy, beans, and isoflavones with risk of cerebral and myocardial infarctions in Japanese populations: the Japan Public Health Center-based (JPHC) study cohort I. Circulation. 2007;116(22):2553-62.
10. Mursu J, Voutilainen S, Nurmi T, Tuomainen TP, Kurl S, Salonen JT. Flavonoid intake and the risk of ischaemic stroke and CVD mortality in middle-aged Finnish men: the Kuopio Ischaemic Heart Disease Risk Factor Study. The British journal of nutrition. 2008;100(4):890-5.
11. McCullough ML, Peterson JJ, Patel R, Jacques PF, Shah R, Dwyer JT. Flavonoid intake and cardiovascular disease mortality in a prospective cohort of US adults. The American journal of clinical nutrition. 2012;95(2):454-64.
12. Zamora-Ros R, Jimenez C, Cleries R, Agudo A, Sanchez MJ, Sanchez-Cantalejo E, et al. Dietary flavonoid and lignan intake and mortality in a Spanish cohort. Epidemiology (Cambridge, Mass). 2013;24(5):726-33.
13. Tresserra-Rimbau A, Rimm EB, Medina-Remon A, Martinez-Gonzalez MA, Lopez-Sabater MC, Covas MI, et al. Polyphenol intake and mortality risk: a re-analysis of the PREDIMED trial. BMC medicine. 2014;12:77.
14. Ivey KL, Hodgson JM, Croft KD, Lewis JR, Prince RL. Flavonoid intake and all-cause mortality. The American journal of clinical nutrition. 2015;101(5):1012-20.
15. Ponzo V, Goitre I, Fadda M, Gambino R, De Francesco A, Soldati L, et al. Dietary flavonoid intake and cardiovascular risk: a population-based cohort study. Journal of translational medicine. 2015;13:218.
16. Ivey KL, Jensen MK, Hodgson JM, Eliassen AH, Cassidy A, Rimm EB. Association of flavonoid-rich foods and flavonoids with risk of all-cause mortality. The British journal of nutrition. 2017;117(10):1470-7.
